# Clinical Profiles and Service Utilisation in Older Adult Psychiatry: An Eight-Year Cohort Study From the United Arab Emirates

**DOI:** 10.1192/bjo.2026.11634

**Published:** 2026-06-30

**Authors:** Syed Ali Bokhari, Tareq Qassem, Syed Fahad Javaid

**Affiliations:** 1 Al Amal Psychiatric Hospital, Emirates Health Services, Dubai, UAE; 2 Maudsley Health, Dubai, UAE; 3 College of Medicine, Mohammed Bin Rashid University of Medicine and Health Sciences, Dubai, UAE; 4 Department of Psychiatry, College of Medicine and Health Sciences, United Arab Emirates University, Al Ain, UAE

## Abstract

**Aims:**

Population ageing in the Gulf Cooperation Council region is accelerating, yet epidemiological data on older adults accessing specialist mental health services remain scarce. This study aimed to describe the demographic profile, diagnostic distribution, psychotropic prescribing patterns, and service utilisation among older adults receiving specialist psychiatric care in the United Arab Emirates (UAE), and to identify predictors of polypharmacy and acute service use.

**Methods:**

A retrospective cohort study was conducted including all patients aged ≥60 years who had contact with a tertiary psychiatric hospital in the UAE between January 2018 and December 2025. Service modalities included outpatient, telehealth, emergency, and inpatient care. Demographic, diagnostic, and prescribing data were extracted from electronic records. Descriptive statistics summarised clinical and service characteristics. Multivariable logistic regression was used to identify predictors of polypharmacy (≥3 concurrent psychotropic medications), inpatient admission and 30-day readmission, while negative binomial regression examined predictors of emergency presentations and overall service utilisation.

**Results:**

The cohort comprised 1,363 patients (mean age 68.7 ± 8.2 years; 57.4% female) representing 55 nationalities. Neurocognitive disorders were the most prevalent diagnosis (31.0%), followed by depressive disorders (23.0%) and schizophrenia spectrum disorders (20.0%). Psychotropic medication was prescribed to 89.8% of patients, with polypharmacy in 58.6%. Second-generation antipsychotics (61.7%) and benzodiazepines (59.6%) were the most frequently prescribed classes. Across the study period, 77.2% of patients received outpatient care, 65.8% used telehealth, 42.3% presented to emergency services and 23.2% had at least one inpatient admission; telehealth accounted for 45.2% of all recorded encounters. Bipolar disorder was the strongest predictor of inpatient admission (adjusted odds ratio [aOR] 6.91, p<0.001) and polypharmacy (aOR 4.88, p<0.001). Substance-related and schizophrenia spectrum disorders were also consistently associated with higher odds of hospitalisation and complex prescribing. Psychiatric comorbidity predicted both emergency presentations (incidence rate ratio 2.64, p<0.001) and 30-day readmission (aOR 2.21, p<0.05). Median inpatient length of stay was 22.6 days.

**Conclusion:**

This large older adult psychiatry cohort from the UAE demonstrates high psychotropic exposure, substantial polypharmacy and significant use of acute services. Bipolar, substance-related and schizophrenia spectrum disorders emerged as key drivers of hospitalisation and complex prescribing. Telehealth constituted nearly half of all clinical encounters, challenging assumptions about low digital engagement among older Arab populations. Priorities for service development include medication optimisation and governance, crisis prevention pathways for high-risk diagnostic groups, and culturally responsive expansion of geriatric telepsychiatry.

No financial sponsorship was received for this project.

